# The development and cognitive testing of the positive outcomes HIV PROM: a brief novel patient-reported outcome measure for adults living with HIV

**DOI:** 10.1186/s12955-020-01462-5

**Published:** 2020-07-06

**Authors:** K. Bristowe, F. E. M. Murtagh, P. Clift, R. James, J. Josh, M. Platt, J. Whetham, E. Nixon, F. A. Post, K. McQuillan, C. Ní Cheallaigh, M. Kall, J. Anderson, A. K. Sullivan, R. Harding

**Affiliations:** 1 King’s College London, Cicely Saunders Institute of Palliative Care, Policy & Rehabilitation, Bessemer Road, London, SE5 9PJ UK; 2 Wolfson Palliative Care Research Centre, Hull York Medical School, University of Hull, Hull, UK; 3 King’s College Hospital NHS Foundation Trust, London, UK; 4 University of Sussex, Brighton, UK; 5 UK-CAB, London, UK; 6 Brighton and Sussex University Hospitals Trust, Brighton, UK; 7 St James’s Hospital Dublin and Trinity College Dublin, Dublin, Ireland; 8 Public Health England, London, UK; 9 Homerton University Hospital NHS Foundation Trust, London, UK; 10 Chelsea and Westminster Hospital NHS Foundation Trust, London, UK

**Keywords:** HIV, Outcomes, PROMs, Implementation, Person-centred

## Abstract

**Background:**

People living with HIV experience burdensome multidimensional symptoms and concerns requiring person-centred care. Routine use of patient reported outcome measures can improve outcomes. There is no brief patient reported outcome measure (PROM) that currently reflects the breadth of concerns for people living with HIV. This study aimed to develop and cognitively test a brief novel patient reported outcome measure for use within routine adult HIV care– the “Positive Outcomes” HIV PROM.

**Methods:**

Development followed the COSMIN taxonomy and guidance for relevance and comprehensiveness, and Rothrock guidance on development of valid patient reported outcome measures. The Positive Outcomes HIV PROM was developed by a steering group (people living with HIV, HIV professionals and health services researchers) using findings from a previously reported qualitative study of priority outcomes for people living with HIV. The prototype measure was cognitively tested with a purposive sample of people living with HIV.

**Results:**

The Positive Outcomes HIV PROM consists of 23 questions (22 structured, and one open question) informed by the priorities of key stakeholders (*n* = 28 people living with HIV, *n* = 21 HIV professionals and *n* = 8 HIV commissioners) to ensure face and content validity, and refined through cognitive testing (*n* = 6 people living with HIV). Cognitive testing demonstrated high levels of acceptability and accessibility.

**Conclusions:**

The Positive Outcomes HIV PROM is the first brief patient reported outcome measure reflecting the diverse needs of people living with HIV designed specifically for use in the clinical setting to support patient assessment and care, and drive service quality improvement. It is derived from primary data on the priority outcomes for people living with HIV and is comprehensive and acceptable. Further psychometric testing is required to ensure reliability and responsiveness.

## Background

People living with HIV experience multidimensional symptoms and concerns that can be burdensome [1]. Despite advances in antiretroviral therapy (ART), people living with HIV have worse health related quality of life than the general population [2], and disproportionate burden of poor mental health [3, 4]. Their physical, cognitive, psychological, social, spiritual, welfare and informational needs are highly interrelated, and persisting HIV stigma can reduce access to appropriate support [5]. A holistic approach to HIV treatment and care is needed, focused on the individual, rather than treating symptoms and concerns in isolation.

Global HIV policy and public health initiatives continue to focus attention on diagnosis, treatment adherence and viral suppression in order to end the HIV/AIDS epidemic [6]. People living with HIV have long reported that this focus on viral suppression and treatment adherence is reflected within clinical consultations, with a lack of attention or opportunity to discuss needs beyond ART-related concerns [5, 7, 8]. Care that focuses on what matters to the individual, and is respectful and responsive to their needs, can improve care experiences and health outcomes [9–11]. Person-centred care incorporating patient reported outcome measures (PROMs) can improve patient-clinician communication, clinical decision-making, symptom recognition and treatment adherence [12, 13]. As well as improving outcomes at an individual level, PROMs can also ensure services deliver equitable and high quality care that meets the needs of their population [14, 15].

Recent European initiatives have promoted a personalised outcomes-focused approach to HIV treatment and care [12, 16, 17]. The routine use of a brief, valid patient-centred PROM which incorporates the breadth of symptoms and concerns experienced by people living with HIV allows an iterative approach to monitoring and optimising care delivery [18, 19]. Administering this PROM before the clinical encounter would enable clinicians to rapidly assess the individual needs of the patient and focus the consultation accordingly. This would exploit the benefits of PROMs in driving and evaluating routine care, as their use is currently largely limited to clinical trials [18]. Importantly, however, use of a PROM also supports a patient-centred approach; enabling patients to be participants in, not just recipients of, their care in line with the BHIVA standards of care [20].

Although quality of life measures have sensitivity to change for people living with HIV [21], there is no tool with established face and content validity to reflect routine HIV treatment and care outcomes. To achieve its potential in person-centred care, the PROM must reflect what matters most to people living with HIV. It must be specific enough to drive a response from health care professionals, rather than measure broader concepts of “quality of life”, which may be more challenging to respond to clinically.

A systematic review of PROMs in HIV trials [18] and a search of the Oxford PROMs database [19] identified HIV PROMs for specific single-dimension outcomes such as depression, stigma, lipodystrophy, adherence, quality of life and self-care, but no brief PROM designed to measure the range of multidimensional outcomes relevant to HIV treatment and care. An updated PubMed search was undertaken for PROMs used in HIV using the Donabedian definition of healthcare quality i.e. we searched for PROMs that aimed to measure a change in health status attributable to preceding health interventions [22]. We applied the WHO definition of health as physical, psychological and social wellbeing [23], and searched for brief tools with items that could reasonably be expected to be responded to under routine HIV care, i.e. was feasible for routine day-to-day practice. No suitable tool was identified.

HIV community groups and professionals have advocated for a new PROM for use within routine HIV care [24]. This study is part of a programme of work to improve assessment and management of symptoms and concerns for people living with HIV. This article presents the development and initial cognitive testing of a novel, brief, patient-centred PROM (the Positive Outcomes HIV PROM) for use in routine adult HIV care that reflects the range of multidimensional outcomes relevant to people living with HIV to drive and evaluate care. This paper build on a previously reported qualitative study of priority outcomes for people living with HIV [5].

## Methods

### Design

Development of the novel Positive Outcomes HIV PROM was undertaken following the COSMIN (COnsensus-based Standards for the selection of health Measurement INstruments) taxonomy and guidance for relevance and comprehensiveness of PROMs to ensure content validity [25, 26], and Rothrock guidance on the development of valid PROMs [27] in three phases (modified from the Rothrock guidance [27]): [1] gathering input from key stakeholders to define concepts and develop a conceptual model [2]; item generation; and [3] item improvement. The methods for each of these three phases are described below.

Following identification of the need for a new PROM, a PROM development steering group was created to guide the process, including: four people living with HIV, four health services researchers and five HIV healthcare professionals (see Table 1).

1. **Gathering Input from key stakeholders**

**Table 1:** PROM Development Steering Group

**People living with HIV (*n*** **=4)**	
Gender	Male (*n* = 3), female (*n* = 1)
Sexual orientation	Gay (*n* = 2), heterosexual (*n* = 2)
Ethnicity	White British (*n* = 4)
**Health Services Researchers (*n*** **= 4)**	
Gender	Male (*n* = 1), Female (*n* = 3)
Expertise	Health services researcher and psychometrician (*n* = 1) Health services researcher, HIV researcher and psychometrician (*n* = 1) Health services researcher and communication scientist (*n* = 1) Epidemiologist and HIV researcher (*n* = 1)
**HIV Professionals (*n*** **= 5)**	
Gender	Male (*n* = 1), female (*n* = 4)
Profession	Doctor (*n* = 4), registered nurse (*n* = 1)

#### In-depth Interviews

The first stage of PROM development was to gather information from key stakeholders, to define concepts and form a conceptual model to underpin the item generation [27], and ensure face and content validity of the new PROM [28]. Adhering to COSMIN principles (recognising the patient as an expert in their condition) [25] interviews were chosen to explore priorities for people living with HIV, as well as their views regarding the design of the PROM. To ensure that the PROM was clinically relevant and would benefit service planning, HIV professionals and commissioners of HIV services were also interviewed regarding priority areas for inclusion, and PROM format. The full methods for the qualitative study of priority outcomes for people living with HIV, which formed the conceptual model to inform the item generation, is published separately [5]. A brief summary of the methods is presented here.

People living with HIV and HIV professionals (clinical and allied health and social care professionals) were recruited from three large teaching hospitals in London (UK), Brighton (UK) and Dublin (Ireland) by clinicians at each site. HIV professionals were also recruited through the British HIV Association via self-referral. Commissioners of HIV services were recruited in England via direct contact. Interviews explored priority outcomes for people living with HIV (previously published, but a summary presented in results here for information) [5], as well as preferred structure, design and frequency of use of an HIV specific PROM (presented here). Interviews were analysed using thematic analysis [29], with a supplementary framework analysis [30] to allow comparison of themes within and across key stakeholder groups (people living with HIV, HIV professionals and HIV commissioners). Further details regarding the purposive sampling frame, inclusion and exclusion criteria, recruitment, conduct of the interviews and analysis are published separately [5].

2. **Item Generation**

The next phase was to generate items for inclusion, aligned to the views of key stakeholders. A two stage item generation process was undertaken.

#### Item generation - stage 1

The first stage consisted of a full day item generation meeting attended by the PROM development steering group. The meeting commenced with presentations providing an overview of PROMs, and their development and use in healthcare more broadly and within HIV care specifically. Following this, the findings from the in-depth interviews with key stakeholders were presented including themes and subthemes from the primary interview data [5] (thematic analysis [29]) and a matrix of thematic saturation across participant groups (framework analysis [30]) to inform discussions of priority items for inclusion. In addition findings from the interview data were presented regarding preferred design and length of the PROM, frequency of use and recall period, and potential utility of the PROM at a patient, service, and commissioning level. Lastly, findings from a national survey of the lives, experiences and healthcare needs of people living with HIV in the UK (Positive Voices) [31] were also reviewed.

Informed by the presentations described above an item generation process was conducted. Discussions were guided by the health services researchers, allowing for free discussion between people living with HIV and the HIV professionals. Informed by the qualitative research findings, discussion commenced with the broad structure of the PROM domains before moving into review of items within each domain. Domains were discussed consecutively and priority items for inclusion identified before moving on to the next domain. At the close of the meeting a summary of the domains and draft items was presented back to the group and consensus agreed.

#### Item generation - stage 2

After the item generation meeting, KB, a communication scientist, reviewed existing measures and drafted questions for each of the selected items for inclusion. The prototype tool was circulated electronically to the PROM development steering group for the second round of the item generation meeting. Minor changes to the wording to improve clarity and comprehension were recommended, and the prototype Positive Outcomes HIV PROM was then finalised and formatted for cognitive testing and item improvement.

3. **Item improvement**

Cognitive interviewing or testing of a tool involves processes of ‘think aloud’ and ‘verbal probing’ to determine the acceptability and accessibility of the format and structure of a tool, interpretation of items, how responses are formulated, and whether any key concepts have been missed [32]. People living with HIV were recruited from two large teaching hospitals in London and Brighton (UK) applying purposive sampling criteria: sexual orientation; ethnicity; and gender. Recruitment continued until thematic saturation was achieved and no new problems or concerns with the Positive Outcomes HIV PROM were emerging from subsequent interviews. It was estimated that 5–10 participants would be required to achieve saturation. Interviews were audio recorded, and hand written notes and comments captured by the researcher (KB). All cognitive interview data were tabulated by item and participant, reviewed by the research team, and consensus reached regarding whether the change should be implemented. The resultant revised Positive Outcomes HIV PROM was circulated to the PROM development steering group and finalised.

## Results

1. **Input from key stakeholders**

### Participants

Fifty-seven key stakeholders were recruited for in-depth qualitative interviews to inform the development of the Positive Outcomes HIV PROM: *n* = 28 HIV patients, *n* = 21 HIV professionals *n* = 8 HIV commissioners. See Table 2 for participant characteristics (previously reported [5]).

**Table 2:** In-depth interview participant characteristics (*n* = 57)

**HIV Patients (*n*** **= 28)**	
Gender	Male (*n* = 14), Female (*n* = 14)
Sexual orientation	Gay (*n* = 10) Heterosexual (*n* = 17) Bisexual (*n* = 1)
Ethnicity	White British (*n* = 12) White Irish (*n* = 8) Black African, Black Caribbean, or Black British (*n* = 8)
Relationship status	Single (*n* = 14) In a relationship (*n* = 14)
Age (years)	Range 23–81, median 45.5
Years since diagnosis	< 5 years (*n* = 7) 6–15 years (*n* = 5) 16–20 years (*n* = 9) > 20 years (*n* = 7)
Comorbidities	None (*n* = 3) 1–2 (*n* = 12) 3 or more (*n* = 13) ^a^
Interview duration (minutes)	Range 13–111, median 53.5
**HIV Professionals (*n*** **= 21)**	
Gender	Male (*n* = 8) Female (*n* = 13)
Profession	Doctors (*n* = 7) Registered nurses (*n* = 7) Allied Health and Social Care Professionals (*n* = 7) ^b^
Interview duration	Range 13–84, median 55 min
**HIV Commissioners (*n*** **= 8)**	
Gender	Male (*n* = 3) Female (*n* = 5)
Role / Employer	NHS (*n* = 4) Local Authority (*n* = 4)
Region	London (*n* = 4) Out of London (*n* = 4)
Regional Prevalence	Very High (*n* = 2) High-Very High [3] Low-Very High [3]
Interview duration	Range 38–69, median 57 min

^a^Comorbidities: Hypertension, diabetes, history of stroke, heart failure, chronic obstructive pulmonary disease, atrial fibrillation, aortic aneurism, history of liver transplant, Hepatitis B, Hepatitis C, renal failure, osteopenia, osteoarthritis, osteoporosis, polyarthropathy, rheumatic arthritis, gout, anaemia, hyperparathyroidism, hyperlipidaemia, gastro-oesophageal reflux disease, coeliac disease, history of bowel cancer, anxiety, depression, personality disorder, bipolar disorder, and drug dependence (on methadone)

^b^Health and social care professionals: welfare officer, psychologist, dietitian, physiotherapist, phlebotomist, and two pharmacists

### Findings

#### PROM structure, design, frequency of use, and content

##### i. PROM length

There was broad consensus across stakeholder groups that people living with HIV were more likely to complete a short PROM. When asked to specify what length of tool would be acceptable, the most frequently delivered responses were ‘no more than two pieces of paper (up to four sides)’, or a maximum of 25 questions.

##### ii. Recall period

Memory problems described in the interviews informed the decision to keep the recall period relatively short, between 2 and 4 weeks. To ensure consistency of the tool across all domains, a recall period of 4 weeks was set for all questions.

##### iii. Response categories

The broad structure of the PROM was designed to include a series of questions with a typical 5-point Likert scale, for ease of use and analysis. However, key stakeholders also recommended the inclusion of a single open-ended question, offering individuals the opportunity to suggest areas for discussion, or raise concerns in addition to those listed in the questions. This was agreed by the PROM development steering group.

##### iv. Frequency of completion

Across stakeholder groups, the majority felt that completion of the PROM at every contact would be most useful. Although there was recognition that this would vary from person to person, as those with more complex issues, and those recently diagnosed, would tend be seen more frequently. At a minimum it was recommended that the PROM should be completed annually.

##### v. PROM domains and items

The in-depth interviews identified six broad domains of need for inclusion within the PROM: physical, cognitive, psychological, social and relational, welfare, and information needs (see previously reported qualitative study of priority outcomes for people living with HIV) [5].

2. **Item generation**

### Participants

Thirteen members of the PROM development steering group: *n* = 4 people living with HIV, n = 4 health services researchers and *n* = 5 HIV healthcare professionals (see Table 1 for participant characteristics).

### Findings

#### Item generation - stage 1

From the conceptual model of priorities, problems and concerns for people living with HIV [5], candidate items were generated and prioritised by the PROM development steering group for inclusion within each of the six domains (Table 3). In addition to the specific questions within each domain, both key stakeholders from the in-depth interviews and the PROM development steering group requested the inclusion of a question to give a global assessment of wellbeing (a sense of ‘where the person is at’).

**Table 3:** Priority items for inclusion identified in item generation meeting

Domain	Priority Items
**Physical**	Pain Stomach and bowel Ability to carry out usual activities / activities of daily living
**Cognitive**	Memory Concentration Sleep
**Psychological**	Anxiety Depression Concerns around disclosure of HIV status Self esteem Spiritual needs / Feeling at peace
**Welfare**	Safety at home and in relationships Recreational drug or alcohol use Money Housing Immigration status
**Social and relational**	Isolation or loneliness Concerns about sexual health, family planning, intimacy, menopause, erectile dysfunction and libido
**Information**	Information needs about managing their HIV
**Global**	Global assessment of wellbeing

There were challenges in the development of items within the domains (Table 3), particularly developing questions which would be appropriate for the heterogeneous population of people living with HIV. It was important that all questions ‘could’ be appropriate to all individuals. For example, questions exploring the importance of religious or spiritual wellbeing within the in-depth interviews, received diverse responses. Some individuals, particularly those from the black African and black Caribbean communities, described the importance of religion and spirituality to them, and its place within a PROM; white British participants, many of whom were gay men, did not always welcome an item about religious or spiritual wellbeing. This underpinned the decision to use the construct ‘at peace’ to explore spiritual and/or existential wellbeing – a phrase which has been adopted in palliative care measures to meet diverse needs beyond those with a faith or religion [33].

Similarly, items related to menopause for women, and erectile dysfunction for men, due to their gender specific nature would not be appropriate for all people living with HIV, and were therefore not included within the PROM. As an alternative, a decision was made to include four items asking more broadly about sex or intimacy, sexual health, contraception and starting a family or having a child. Provision of response options from ‘not at all’ to ‘always’ for all questions (except the global assessment of wellbeing and information needs questions, which have different response categories) made it possible for every individual to answer every question within the response categories provided, without the need for an additional ‘not applicable’ option. By including broader items it is expected that these will create an opportunity for more specific concerns to be raised in the consultation.

#### Item generation - stage 2

A 23 item prototype PROM was developed, with initial introductory text and brief sentences to explain the shifts between domains. Questions about cognitive function were placed within the domain of ‘physical’ to avoid confusion around the term ‘cognitive’.

The prototype Positive Outcomes HIV PROM was further refined by the PROM development steering group. Revisions at this stage included minor editorial changes to simplify the language in places, including replacing the word ‘concern’ with ‘worry’ and replacing ‘consider’ with ‘think about’. In addition, the word ‘your’ was added into some questions where there was potential for ambiguity (‘have you been worried about YOUR sexual health’). Lastly, scale direction was amended where necessary to enable ease of interpretation through a total score. The PROM was then finalised for cognitive testing and item improvement.

3. **Item improvement**

### Participants

Six people living with HIV were recruited for cognitive testing of the PROM (see Table 4 for details).

**Table 4:** Cognitive testing participants

People living with HIV (***n*** = 6)	
Gender	Male (*n* = 4) Female (*n* = 2)
Sexual orientation	Gay (*n* = 3) Heterosexual (*n* = 3)
Ethnicity	White British (*n* = 4) Black African, Black Caribbean, or Black British (*n* = 2)
Relationship status	Single (*n* = 1) In a relationship (*n* = 5)
Age	Median 51 years (range 40–57)
Interview duration	Median 52 min (range 32–120)

### Findings

i. *Cognitive testing and item improvement*

Cognitive testing of the new PROM demonstrated high levels of acceptability, with no suggested changes to the structure, included questions or question order. Minor revisions to the wording were proposed to improve the clarity and comprehension of items, including: adding an explanation for neuropathy (‘which might include pins and needles or burning pain’); addition of ‘joint pain’ to the list of example pains described; addition of ‘your’ to two more questions ‘worries about YOUR housing’ and ‘worries about YOUR immigration status’ (particularly to avoid confusion about immigration as a societal rather than individual concern); and the question which had asked about ‘social support’ was changed to ‘support from people around you’ to avoid confusion with support provided by government or social services.

ii. *Finalisation*

**Fig. 1:**
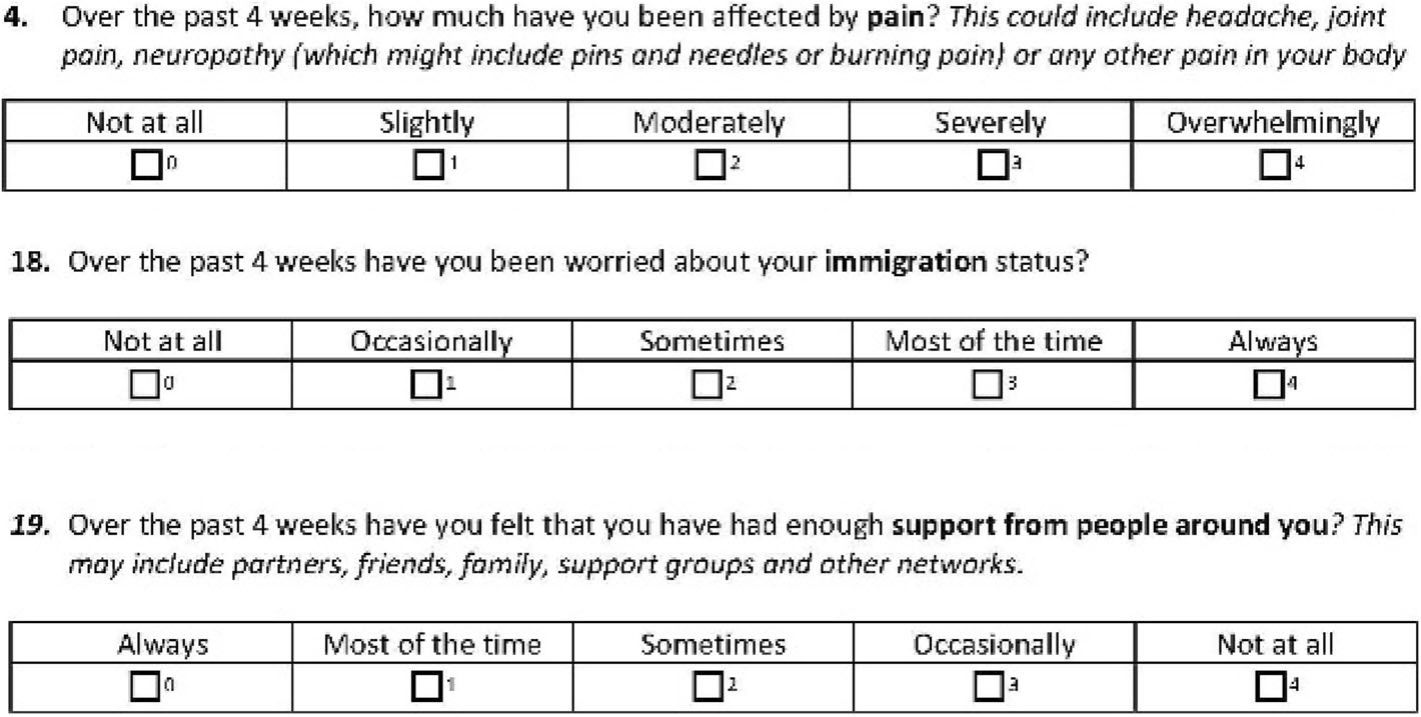
Example items from the Positive Outcomes HIV PROM

The final version of the PROM (Version 1: 30.1.18) that includes 23 items was circulated to the PROM development steering group and agreed (see example questions in Fig. 1 below).

The PROM opens with one open-ended question offering the individual the opportunity to report their three main priorities for focus within their HIV consultation; and 22 questions with standard Likert (five point) response scales. The first of these is a global assessment of wellbeing, and the remaining 21 ask about specific constructs within the 6 domains of need (see Table 5 for a list of domains and summary items).

**Table 5:** Positive Outcomes PROM domains and summary items

Item Number	Domain	Item
1	Priorities	Main problems and concerns (freetext)
2	Global assessment	General health and wellbeing
3	Information	Information needs
4	Physical	Pain
5		Stomach or bowel
6		Memory or concentration
7		Sleep
8		Usual activities
9	Psychological	Anxiety or worry
10		Depression or low mood
11		Telling someone about your HIV status
12		Feeling good about yourself
13		Feeling at peace
14	Social	Safety in relationships
15		Drug or alcohol use
16		Money
17		Housing
18		Immigration
19		Support from people around you
20	Relational	Sex or intimacy
21		Sexual health
22		Contraception
23		Starting a family or having a child

The completed PROM therefore highlights three priority areas for discussion within the consultation, and gives a score of 0–88 for problems and concerns (the higher the score, the higher the burden of symptoms and concerns).

Assessment of the Positive Outcomes HIV PROM against the new COSMIN criteria for evaluating the content validity of PROMs, provides supporting evidence for the relevance, comprehensiveness and comprehensibility (content validity) of the Positive Outcomes HIV PROM [25, 26] (see Table 6).

**Table 6:** COSMIN criteria and rating system for evaluating the content validity of PROMs [26]

Criteria	Assessment
**Relevance**	
Are the included items relevant for the construct of interest?	**✓**
Are the included items relevant for the target population of interest?	**✓**
Are the included items relevant for the context of use of interest?	**✓**
Are the response options appropriate?	**✓**
Is the recall period appropriate?	**✓**
**Comprehensiveness**	
Are all key concepts included?	**✓**
**Comprehensibility**	
Are the PROM instructions understood by the population of interest as intended?	**✓**
Are the PROM items and response options understood by the population of interest as intended?	**✓**
Are the PROM items appropriately worded?	**✓**
Do the response options match the questions?	**✓**

## Discussion

Following the COSMIN taxonomy and guidance for relevance and comprehensiveness, and Rothrock guidance on the development of valid PROMs, a brief novel PROM for adults living with HIV was developed which reflects the breadth of symptoms and concerns for people living with HIV, and the priorities of HIV professionals and commissioners of HIV services [25, 26]. Informed by the views of key stakeholders, the Positive Outcomes HIV PROM has face and content validity [28]. Following cognitive testing [32], the PROM was found to be highly acceptable to people living with HIV, and was refined to improve clarity and comprehension of items.

The development of the Positive Outcomes HIV PROM, a brief, comprehensive tool for use within routine HIV care, represents a significant step towards a personal outcome approach. Pressures on HIV services, and public health and policy drivers [6], arguably privelege a focus on diagnosis and treatment adherence rather than other health and social care needs [5]. The need for person-centred care, focusing on what matters to people living with HIV, has long been recognised [16]. However, to date there was no brief PROM that reflected the breadth of concerns for people living with HIV. Incorporating the Positive Outcomes HIV PROM into routine HIV care will support people living with HIV to set the priorities for their consultations (focusing the care on what matters most to each individual), and enable them to be participants, not just recipients, in their care in line with the BHIVA standards of care for people living with HIV (2018) [20]. The Positive Outcomes HIV PROM will facilitate the standards being achieved and audited over time.

The successful development of the Positive Outcomes HIV PROM was achieved through the co-design and management of the project between health services researchers, people living with HIV and HIV professionals. This means that the tool both reflects the needs and expressed priorities of people living with HIV (ensuring face and content validity) [25], and also the priorities of HIV professionals and commissioners to ensure that it can fit within care services, complement existing care systems and approaches, and inform service development and delivery [5]. Importantly, the design and structure of the tool also reflects the preferences of key stakeholders, improving the potential utility of the Positive Outcomes HIV PROM to be implemented in routine practice.

### Strengths and limitations

A major strength of this study is the methodological rigour with which it was undertaken, with transparent reporting of the PROM development process following both COSMIN and Rothrock guidance [25–27]. Many reports of PROM development fail to describe the processes of item generation in detail. Another strength is the meaningful engagement and inclusion of people living with HIV as experts throughout the study; they advocated for a new HIV PROM, were research participants, and members of the PROM development steering group. The inclusion of interviews with commissioners of HIV services will ensure the PROM is not only relevant to HIV service users and professionals, but also to those who plan and fund HIV care services. Although we worked to ensure that the participants in the in-depth interviews and cognitive interviews represented the diversity (ethnicity, sexual orientation, gender, duration since diagnosis and presence of comorbidities) of people who use HIV services, there was underrepresentation of minority communities outside of black African, black Caribbean and black British people and of trans participants. However, the sample was strengthened by the inclusion of Dublin as a recruitment site. Through targeted initiatives they have successfully engaged those who historically may struggle to access HIV services, including those with unstable HIV, complex social situations, and a history of, or current, drug problems. Lastly, although a breadth of ages was achieved in the in-depth interview sample, for the cognitive interviews this was narrower. This is a potential limitation of the initial cognitive testing, however in subsequent psychometric testing a more representative sample has been sought.

### Future perspectives

Next steps for the validation of the PROM will include implementation through test sites, completion of psychometric evaluation to ensure reliability and responsiveness, and development of a user manual to guide use and implementation according to usual clinical practice. Consideration will also be given at this time to the scoring system for the PROM and any modifications that may be required. In addition, further work to undertake translation and cultural adaption of the PROM will be required to broaden its use internationally. The remaining steps of validation have been undertaken in five European countries (manuscript in preparation). We have provided a list of domains and summary items and the full measure will be freely available once the quantitative elements of the testing have been published.

## Conclusion

The Positive Outcomes HIV PROM is a brief outcome measure that reflects the breadth of symptoms and concerns experienced by people living with HIV, developed in line with recognised international methodological standards. Informed by the priorities of key stakeholders, the Positive Outcomes HIV PROM has supporting evidence for face and content validity and high levels of acceptability following initial cognitive testing. HIV community groups and professionals have advocated for a new PROM for use within routine HIV care. The Positive Outcomes PROM reflects the range of multidimensional outcomes relevant to people living with HIV to drive and evaluate their care.

## Data Availability

The datasets generated and/or analysed during the current study are available from the corresponding author on reasonable request.
